# An uncharacterized protein NY1 targets EAT1 to regulate anther tapetum development in polyploid rice

**DOI:** 10.1186/s12870-022-03976-0

**Published:** 2022-12-13

**Authors:** Nabieu Kamara, Zijun Lu, Yamin Jiao, Lianjun Zhu, Jinwen Wu, Zhixiong Chen, Lan Wang, Xiangdong Liu, Muhammad Qasim Shahid

**Affiliations:** 1 State Key Laboratory for Conservation and Utilization of Subtropical Agro-Bioresources, South China Agricultural University, Guangzhou, 510642 China; 2 Guangdong Laboratory for Lingnan Modern Agriculture, Guangzhou, 510642 China; 3 Guangdong Provincial Key Laboratory of Plant Molecular Breeding, South China Agricultural University, Guangzhou, 510642 China; 4 College of Agriculture, South China Agricultural University, Guangzhou, 510642 China; 5 Sierra Leone Agricultural Research Institute (SLARI), Freetown, PMB 1313 Sierra Leone

**Keywords:** Meiosis, Pollen sterility, Tapetum, Tetraploid rice, RNA-seq

## Abstract

**Background:**

Autotetraploid rice is a useful germplasm for the breeding of polyploid rice; however, low fertility is a major hindrance for its utilization. Neo-tetraploid rice with high fertility was developed from the crossing of different autotetraploid rice lines. Our previous research showed that the mutant (*ny1*) of *LOC_Os07g32406* (*NY1*), which was generated by CRISPR/Cas9 knock-out in neo-tetraploid rice, showed low pollen fertility, low seed set, and defective chromosome behavior during meiosis. However, the molecular genetic mechanism underlying the fertility remains largely unknown.

**Results:**

Here, cytological observations of the *NY1* mutant (*ny1*) indicated that *ny1* exhibited abnormal tapetum and middle layer development. RNA-seq analysis displayed a total of 5606 differentially expressed genes (DEGs) in *ny1* compared to wild type (H1) during meiosis, of which 2977 were up-regulated and 2629 were down-regulated. Among the down-regulated genes, 16 important genes associated with tapetal development were detected, including *EAT1, CYP703A3, CYP704B2, DPW, PTC1, OsABCG26, OsAGO2, SAW1, OsPKS1, OsPKS2,* and *OsTKPR1*. The mutant of *EAT1* was generated by CRISPR/Cas9 that showed abnormal tapetum and pollen wall formation, which was similar to *ny1*. Moreover, 478 meiosis-related genes displayed down-regulation at same stage, including 9 important meiosis-related genes, such as *OsREC8, OsSHOC1, SMC1, SMC6a* and *DCM1*, and their expression levels were validated by qRT-PCR.

**Conclusions:**

Taken together, these results will aid in identifying the key genes associated with pollen fertility, which offered insights into the molecular mechanism underlying pollen development in tetraploid rice.

**Supplementary Information:**

The online version contains supplementary material available at 10.1186/s12870-022-03976-0.

## Background

Autotetraploid rice is derived from diploid rice by doubling its chromosomes. Compared with diploid rice, autotetraploid rice exhibited strong biological advantages, high stress resistance, and high heterosis [1–7]. However, autotetraploid rice displayed complex reproductive defects, including abnormal pollen development and embryo sac, embryogenesis, endosperm development, as well as double fertilization [1, 4, 8–14]. One of the most common defects in autotetraploid rice is the high frequency of abnormal chromosome behavior in pollen mother cells (PMC) during meiosis [9, 13]. Main meiotic defects that lead to autotetraploid sterility include chromosome straggling during metaphase I and metaphase II, and chromosome lagging during anaphase I and anaphase II [9, 13]. In contrast, little abnormal chromosomal behavior during meiosis, and large numbers of differentially expressed genes in meiotic anthers were detected in neo-tetraploid rice relative to autotetraploid lines [15–19]. Neo-tetraploid rice lines exhibited high yield potential, unique structural classification and a novel specific allele associated with heat tolerance [20, 21]. Previous studies detected at least four genes, including *MOF1*, *OsMND1*, *NY1* and *NY2* that may associate with pollen fertility in tetraploid rice [18, 22, 23]. However, the molecular mechanism of these genes underlying pollen fertility process remained largely unknown in tetraploid rice.

A flowering plant's anther contains both reproductive and non-reproductive tissues. After morphogenesis, each anther lobe contains microsporocyte, tapetum, epidermis, endothecium, and middle layer. An anther's tapetum is the innermost cell layer that provides a safe environment, enzymes, and nutrients for microspore development [24]. *PTC1* encodes a PHD-finger protein that cause microspore abortion, and control pollen wall formation and tapetal degeneration [25]. The meiotic process is vital for pollen development, and more than 5000 genes regulate meiosis [26–35].

Transcriptome analysis can provide valuable insights into rice pollen development by detecting gene regulation. The TIGR (The Institute for Genomic Research Rice Genome Annotation) and GEO (Gene Expression Omnibus) provide public access to a number of transcriptome datasets [36, 37]. The characteristics of genetic regulation in pollen development have been analyzed using reliable pollen development networks and novel pollen development-related genes. Several studies have examined the pollen development process in autotetraploid rice [9, 12, 13]. According to Guo et al. [15], significant differences between neo-tetraploid rice and its two parents were detected, and 42 meiosis-specific genes were identified by RNA sequence analysis. During the meiosis stage of autotetraploid rice T449-4x, 75 meiosis-related genes displayed differential expressions compared with diploid rice [12]. A total of 122 genes were discovered in an autotetraploid rice line (02,428-4x) that could be linked to low pollen fertility during pollen formation [13]. RNA sequencing revealed that polyploidy increased multi-allelic interactions at pollen sterility loci and increased chromosomal abnormalities in autotetraploid rice [10]. Moreover, small RNA sequencing revealed that particular differentially expressed miRNAs in autotetraploid rice produced partial embryo sac and pollen sterilities [11, 38]. The genetic regulation of normal pollen fertility in neo-tetraploid rice might be revealed by using all of these RNA sequencing data. In tetraploid rice mutants, however, information about genetic regulation of pollen fertility is limited.

In our previous study, loss-of-function of *NY1* (*LOC_Os07g32406*) mutant (*ny1*) displayed low seed set, low pollen fertility and high frequency of straggled chromosomes during meiosis. In this study, we used cytological observations to examine the anther development differences between the wild type (H1) and *ny1* mutant, and RNA-seq was used to analyze the differentially expressed genes (DEGs) between *ny1* and H1 during pollen mother cell meiosis. Moreover, CRISPR/Cas9 was employed to edit *NY1* related downstream gene, *EAT1,* in neo-tetraploid rice line H1 and qRT-PCR was used to validate the important DEGs related to tapetum development and meiosis. The results of this study provide insights into the molecular mechanism underlying pollen development in tetraploid rice.

## Results

### DNA variations, sequencing and cluster analysis of *NY1* (LOC_Os07g32406)

According to MSU7 reference genome, the full length of *NY1* gene is 4429 nucleotides, and its CDS sequence is 1770 bp. In order to analyze the DNA variations in the sequence of *NY1* in different rice materials, the resequencing data of 121 rice lines, including diploid, autotetraploid and neo-tetraploid rice, were analyzed. Twenty-two types of mutations were detected in these materials, with a total of 11mutant sites in CDS (Table S1-S3-1 to S3-22; Figure S1). Two missense mutations were found in *NY1*, which were mutant 1 and 5 (Table S3-1-S3-22; Figure S2). Cluster analysis revealed that most of the neo-tetraploid rice lines and their one parent, T45, clustered together, suggesting that high fertility gene may come from T45 (Figure S3). To investigate the evolutionary relationship between *NY1* and its homologs, BLASTP from the NCBI was used with the *NY1* sequence as a query. High similarity was detected with *japonica* rice (UniRef100_Q8H3F8, 93.22%, 516 bp, Os07g0507500 protein), *indica* rice (UniRef100_B8B6G4, 92.64%, 516 bp and Putative uncharacterized protein), *Arabidopsis thaliana* (UniRef100_Q8LFH9, 45.53%, 380 bp and Putative uncharacterized protein), and *Populus trichocarpa* (UniRef100_B9GTG6, 45.6%, 364 bp and Predicted protein).

### Phenotypic analysis of genetic populations

**Fig. 1:**
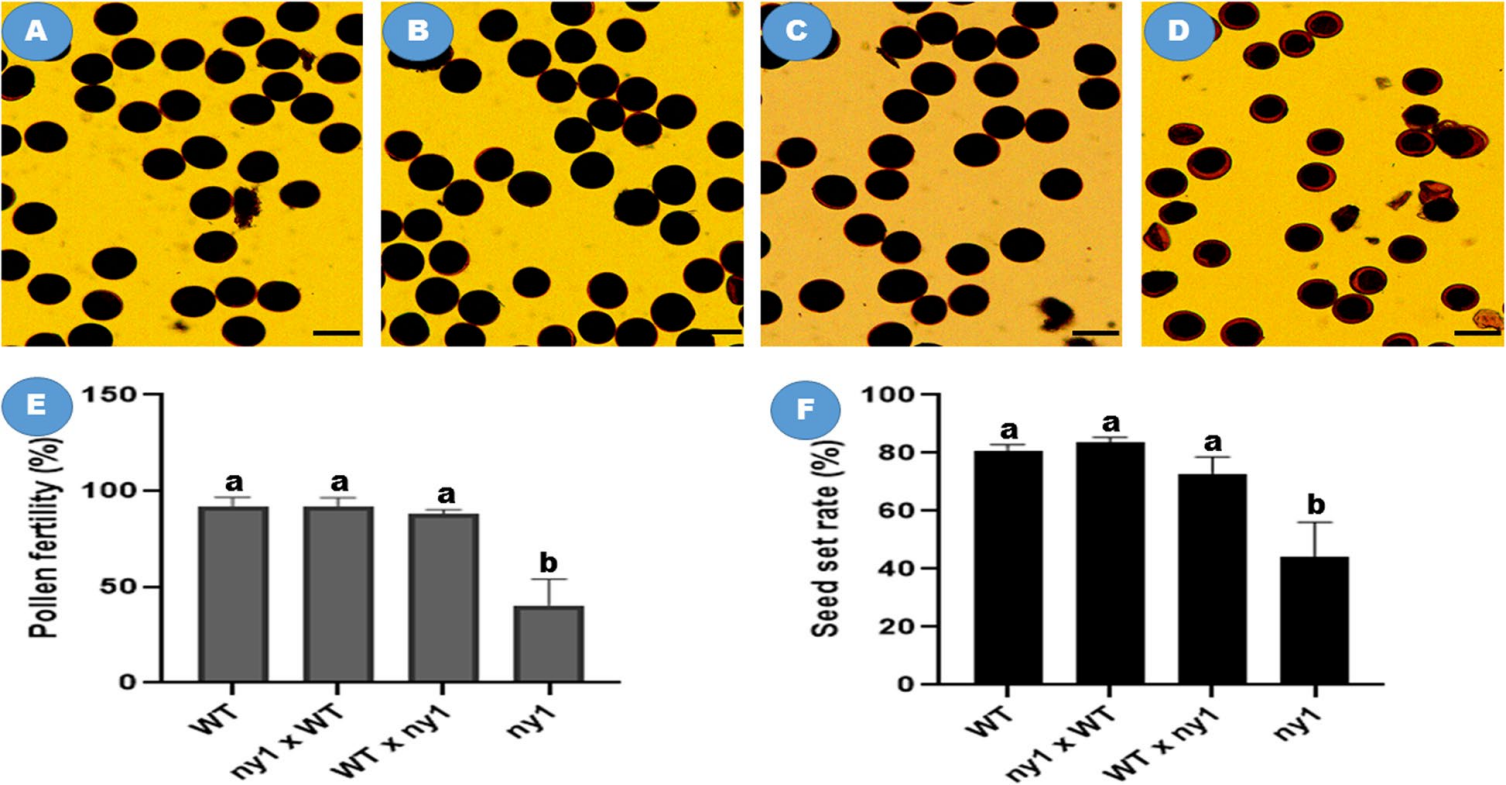
Pollen fertility and seed set in H1 (WT), F_1_ hybrids and *ny1* mutant. A-D indicated pollen grains of WT **A**, *ny1* × WT **B**, WT × *ny1* **C**, and *ny1* **D**, respectively. Bar = 30 μm; E and F represent pollen fertility and seed setting of WT, *ny1* and their F_1_ hybrids, respectively. Ten plants were randomly selected to observe pollen fertility and seed set. Least significant difference (LSD) was used in the multiple comparison tests for each trait. Different letters between two samples indicate significant differences (*p* value < 0.05). Error bars represent SD

In our previous study, *NY1* showed abnormal pollen fertility and high frequency of chromosome behavior abnormalities. As a consequence, seed set rate in the mutant were markedly reduced compared to H1 (the wild type) [23]. In order to explore the reproductive roles of *NY1*, reciprocal crosses were made between *ny1* and H1. Phenotypic analysis indicated that all F_1_ plants from the crosses displayed wild-type phenotypes. Similar to H1 (Fig. 1 A), I_2_-KI staining assay showed that the anthers of all the F_1_ plants were fertile (Fig. 1 B, C) and completely different from *ny1* plants (Fig. 1 D). In comparison with the F_1_ hybrids in both crosses, there were no significant difference between the H1 (WT) and F_1_ hybrids for pollen fertility and seed set, suggesting the dominance of H1 for the fertility trait in both crosses (Fig. 1 E, F). In F_2_ populations, the phenotypes of all progenies from each population were investigated, and mutants displayed abnormal pollen fertility and low seed set (Table S4).

### Cytological comparison of anther development between H1 and *ny1* mutant

**Fig. 2:**
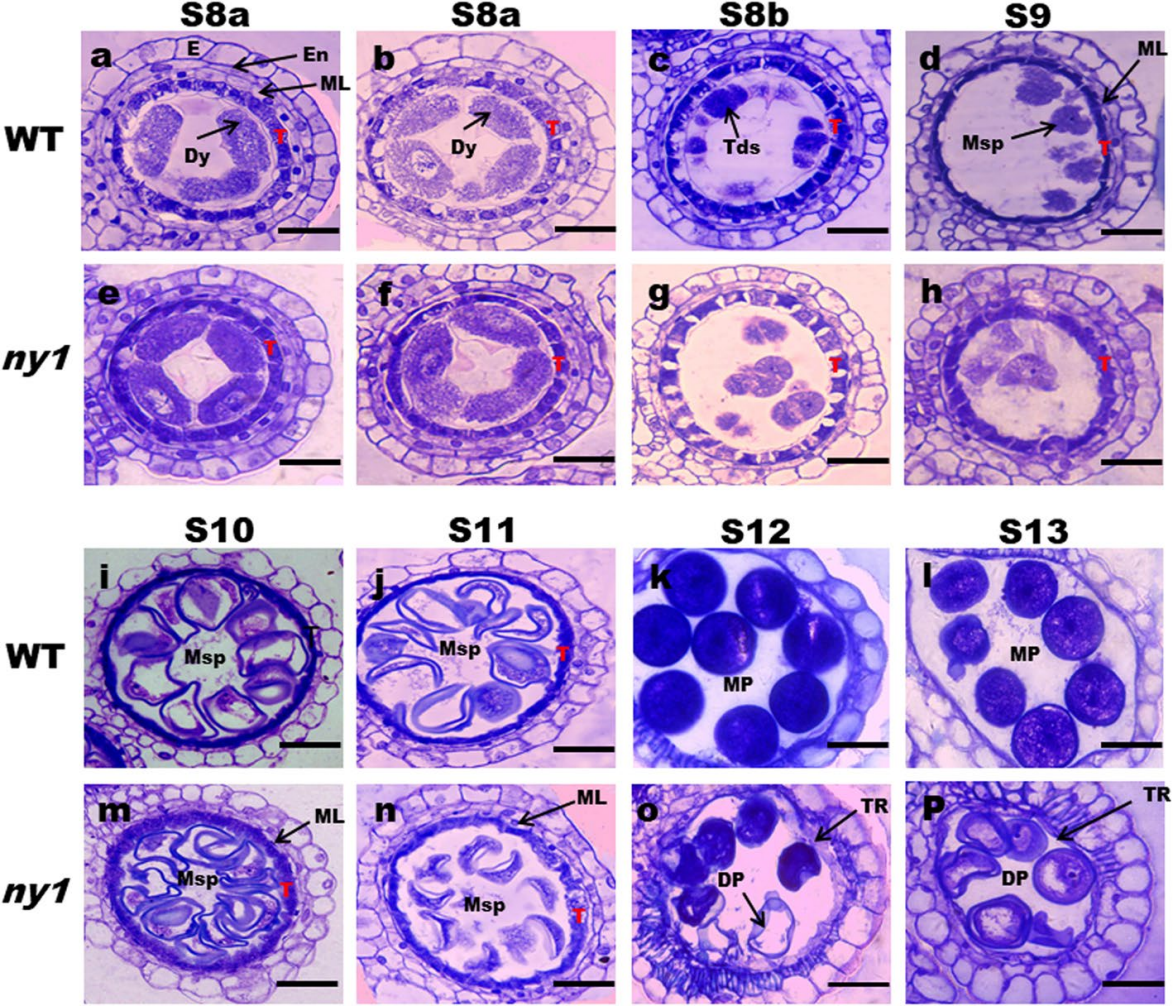
Comparison of anther development differences between the H1 and *ny1* mutants. Stage 8a (a, e), Stage 8a (b, f), Stage 8b (c, g), Stage 9 (d, h), Stage 10 (i, m), Stage 11 (j, n), Stage 12 (k, o), and Stage 13 (l, p). E, epidermis; En, endothecium; ML, middle layer; T, tapetum; PMC, pollen mother cells; Dy, dyad cells; Tds, tetrads; Msp, microspore; MP, mature pollen; DP deformed pollen; TR, tapetal remnants. Bars = 20 μm (a–p)

To investigate the cytological defects and abortion stage during anther development in *ny1*, semi-thin sections of the H1 (WT) and *ny1* mutant anthers at different development stages were examined according to Lu et al. [18] and Mondol et al. [39]. The *ny1* anthers generated morphologically normal pollen mother cells (PMCs) and somatic layers (namely epidermis, endothecium, middle layer, and tapetum) at S8a to S9, just as H1 anthers. However, the tapetum cells in H1 displayed degeneration and became less vacuolated from S8a to S9 stage. At S10 stage, the tapetum cells in H1 were severely shrunken and stained darkly, the middle layer disappeared, and the microspores vacuolated with less cytoplasm contents and formed round shape. In contrast, the *ny1* tapetum cells were also shrunken, but the extent of their shrinkage was far less than that of the H1 tapetum cells and the middle layer remained visible. At the S11 stage, the H1 microspores maintained a defined shape, tapetum became less vacuolated. However, at this stage, *ny1* exhibited condensed tapetum, and the microspores were less stained and lack of sporopollenin at stage 11. Besides this, disruption of mutant microspores was more pronounced and the middle layer still remained visible at S11. At stage 12 and 13, the H1 anther developed and produced spherical, densely stained pollen grains full of starch and lipid. In contrast, the pollen grains of *ny1* were shrunken, empty and surrounded with little debris or residual tapetum remnants (TR) (Table 1; Fig. 2).

**Table 1:** Frequency of pollen mother cells with abnormal middle layer degeneration during meiosis in WT (H1) and *ny1*

	WT		*ny1*	
Stage	Number	Abnormal	Number	Abnormal
S10	31	0.00	28	95.24
S11	49	9.09	44	92.31

S10 and S11 indicate anther development stages. S10 represents the single microspore stage, while S11 represents the late bicellular stage

### Identification and analysis of differentially expressed genes (DEGs) in NY1 compared with H1 (WT) during PMC meiosis

Transcriptome sequencing was done at meiosis stage in *ny1* and H1, and clean data was obtained for further analysis. After the removal of low-quality reads, an average of 51,946,550 high-quality clean reads were obtained from each sample, accounting for 92.53% of the 56,140,313 total reads. Clean reads of each sample were aligned with the reference genome, and the alignment efficiency ranged from 91.49 to 92.69% between *ny1* and H1. The Q30 base percentage in all samples ranged from 93.50% to 93.86%, and the GC content was 49.96% or higher (Table S5). There was a high degree of correlation coefficients among three biological replicates of RNA-seq data between *ny1* and H1 with a Pearson correlation coefficient of more than 0.8436, indicating that the three replications were consistent (Table S6).

**Fig. 3:**
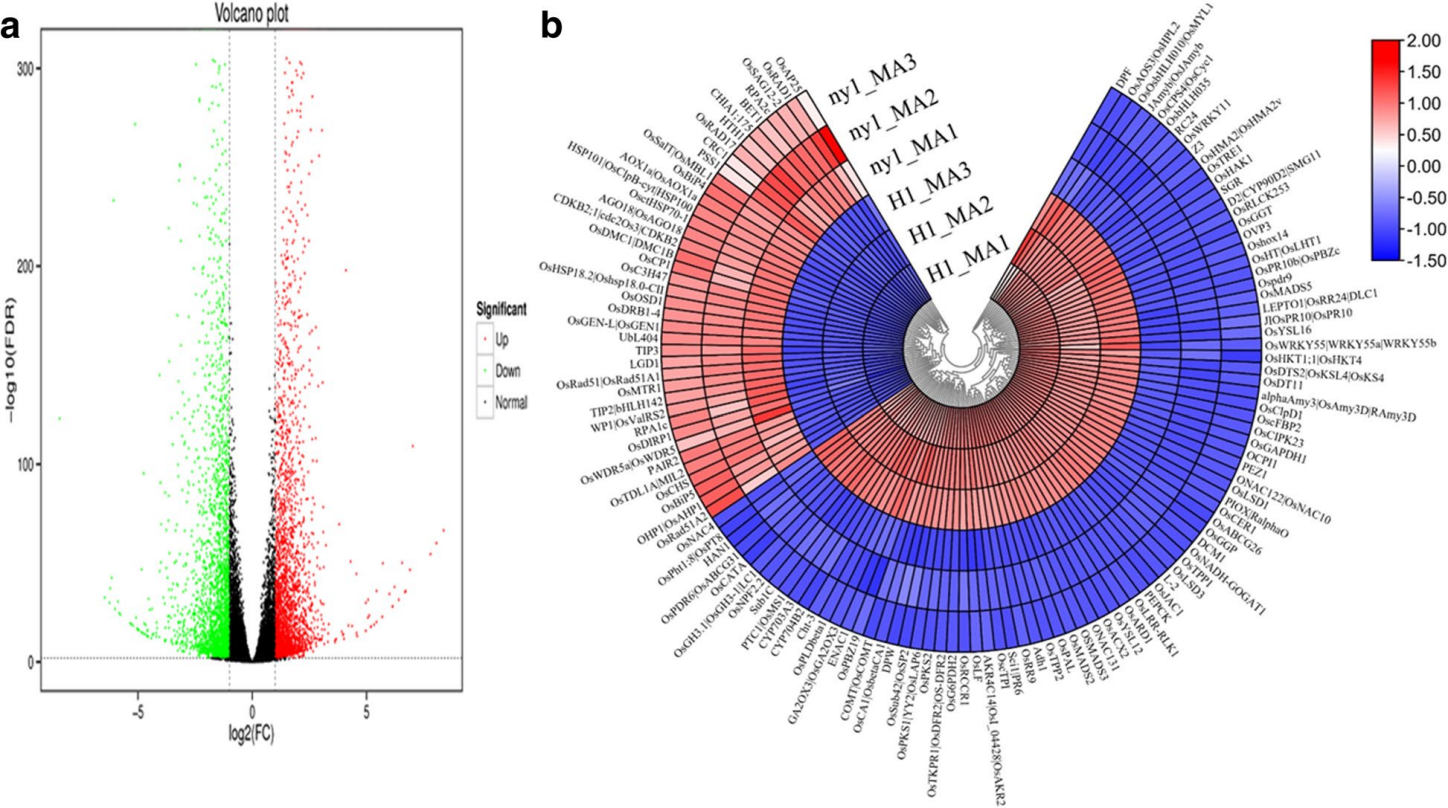
Volcano plot and sliding window plots of the differential gene expression levels between *ny1* and WT. *P* < 0.05 was used as the threshold to judge the significance of difference in gene expression. **a** Differentially expressed genes (DEGs) between *ny1* and H1. **b** The sliding window plots showing the DEGs expression patterns of the three replicates between *ny1* and H1

A total of 5606 differentially expressed genes (DEGs) were obtained between *ny1* and H1 (Fig. 3a; Table S5). Among these DEGs, 2977 were up-regulated and 2629 were found to be down-regulated (Fig. 3a; Table S7). Among the down regulated DEGs, four known genes related to tapetum development, including *EAT1* (*Os04g0599300*)*, OsABCG26* (*Os10g0494300*)*, PTC1* (*Os09g0449000*) *and OsAGO2* (*Os04g0615700*) showed significantly high expression levels between *ny1* and H1 (Table S7). Many important DEGs were highly expressed in H1 when compared to *ny1* mutant (Fig. 3b).

### Gene Ontology (GO) enrichment analysis of DEGs between *ny1* and H1

**Fig. 4:**
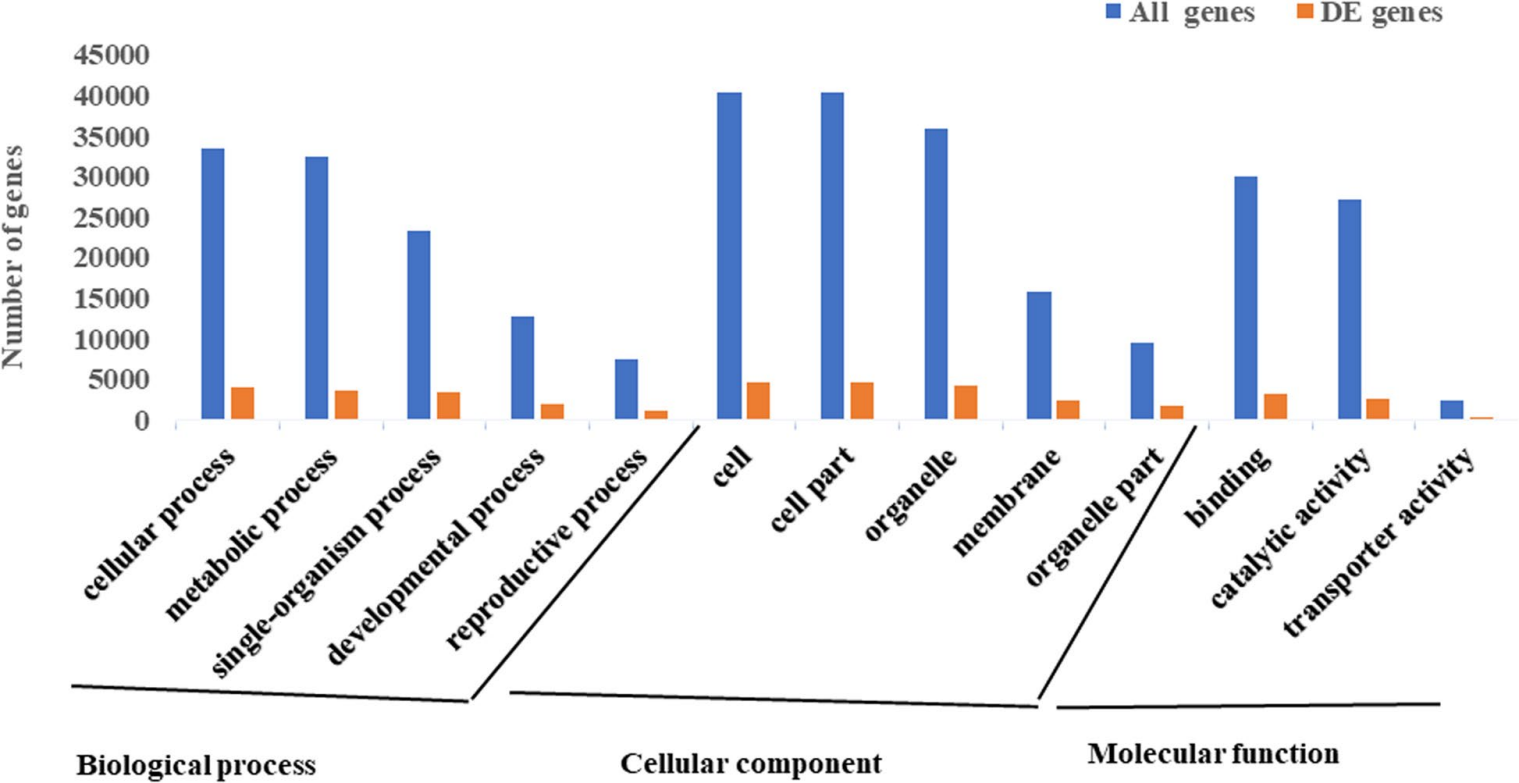
GO analysis of differentially expressed genes classification into three main categories, biological process, molecular function and cellular component. The y-axis indicates the number of DEGs in a category. In the three main GO categories between *ny1* and H1 (WT), top 3 ~ 5 categories related to pollen development were selected

To further characterize the function of the DEGs, GO enrichment analysis was performed on the DEGs obtained between *ny1* and H1. The results showed that all predicted rice genes were assigned to different functional categories. In total, 1241 of the 5606 DEGs between *ny1* and H1 were assigned to at least one GO term in biological process, molecular function, and cellular component categories. Transcripts were further classified into the top13 functional subcategories related to anther or pollen development (Fig. 4). In the biological process category, cellular process and metabolic process were the most significant groups, indicating that the rice anthers during meiosis have wide metabolic activities (Fig. 4).

Further, gene ontology (GO) analysis showed that 119 GO terms were significantly enriched in the up and down-regulated DEGs, respectively. In the biological processes category, 75 GO terms were significantly enriched in the up- DEGs, including photosynthesis (GO:0015979), microtubule-based process (GO:0007017), microtubule-based movement (GO:0007018), translation (GO:0006412), gene expression (GO:0010467), protein metabolic process (GO:0019538), cell cycle process (GO:0022402), cell cycle (GO:0007049), transcription (GO:0006350), and transcription, DNA-dependent (GO:0006351), and 59 GO terms, such as pollen-pistil interaction (GO:0009875), recognition of pollen (GO:0048544), pollination (GO:0009856), reproductive process (GO:0022414), reproduction (GO:0000003), disaccharide metabolic process (GO:0005984), carbohydrate biosynthetic process (GO:0016051), cellular carbohydrate biosynthetic process (GO:0034637), oligosaccharide metabolic process (GO:0009311), carbohydrate metabolic process (GO:0005975), cellular lipid catabolic process (GO:0044242), cellular lipid metabolic process (GO:0044255), cell death (GO:0008219), and programmed cell death (GO:0012501), were enriched in the down-regulated genes (Tables 2 and 3; Table S8a, b). In the molecular function category, 3 GO terms, including structural constituent of ribosome, structural molecule activity and rRNA binding, were identified to be up-regulated and 58 GO terms including purine nucleoside binding, adenyl nucleotide binding, nucleoside binding, adenyl ribonucleotide binding and ATP binding were found in the down-regulated DEGs. In the cellular component category, a total of 41 and 2 GO terms were identified to be significantly enriched in the up and down-regulated DEGs, respectively (Figure S4, S5, S6, S7; Table S8a, b). All of these results indicated that these DEGs were enriched in key biological processes associated with anther or pollen development in the down-regulated genes (Table 3; Table S8b).

**Table 2:** Significant GO terms of up-regulated differentially expressed genes in the biological process category between *ny1* and H1 (WT) during meiosis

GO_term	GO_term_annotation	*p*-value	FDR
GO:0010467	gene expression	2.3E-60	2E-57
GO:0044267	cellular protein metabolic process	3.5E-38	2E-35
GO:0019538	protein metabolic process	6.4E-17	2.7E-14
GO:0016070	RNA metabolic process	4E-07	0.000076
GO:0007049	cell cycle	1.7E-06	0.00022
GO:0044249	cellular biosynthetic process	0.000016	0.0017
GO:0009058	biosynthetic process	0.000034	0.0035
GO:0019222	regulation of metabolic process	0.000058	0.0046
GO:0006350	transcription	0.000073	0.0046
GO:0022402	cell cycle process	0.000067	0.0046
GO:0007018	microtubule-based movement	0.000069	0.0046
GO:0044257	cellular protein catabolic process	0.0001	0.006
GO:0031323	regulation of cellular metabolic process	0.00013	0.0064
GO:0010468	regulation of gene expression	0.00012	0.0064
GO:0007017	microtubule-based process	0.00018	0.0069
GO:0080090	regulation of primary metabolic process	0.0002	0.0073
GO:0045449	regulation of transcription	0.00064	0.02
GO:0015979	photosynthesis	0.00092	0.026
GO:0009987	cellular process	0.0011	0.031
GO:0006351	transcription, DNA-dependent	0.0014	0.036

**Table 3:** Significant GO terms of down regulated differentially expressed genes in the biological process category between *ny1* and H1 (WT) during meiosis

GO_term	GO_term_annotation	*p*-value	FDR
GO:0012501	programmed cell death	3E-28	2.4E-25
GO:0008219	cell death	5.7E-28	2.4E-25
GO:0044267	cellular protein metabolic process	1.1E-09	1.3E-07
GO:0006810	transport	2.5E-07	0.000024
GO:0009875	pollen-pistil interaction	9.8E-07	0.000066
GO:0048544	recognition of pollen	9.8E-07	0.000066
GO:0009856	pollination	9.8E-07	0.000066
GO:0022414	reproductive process	9.8E-07	0.000066
GO:0000003	reproduction	0.000011	0.00067
GO:0005984	disaccharide metabolic process	0.0002	0.0096
GO:0016137	glycoside metabolic process	0.0002	0.0096
GO:0016051	carbohydrate biosynthetic process	0.00032	0.015
GO:0009311	oligosaccharide metabolic process	0.00033	0.015
GO:0044242	cellular lipid catabolic process	0.00037	0.016
GO:0044255	cellular lipid metabolic process	0.00038	0.016
GO:0034637	cellular carbohydrate biosynthetic process	0.00039	0.016
GO:0005975	carbohydrate metabolic process	0.0017	0.049

### Kyoto Encyclopedia of Genes and Genomes (KEGG) pathway enrichment analysis of DEGs between *ny1* and H1

**Fig. 5:**
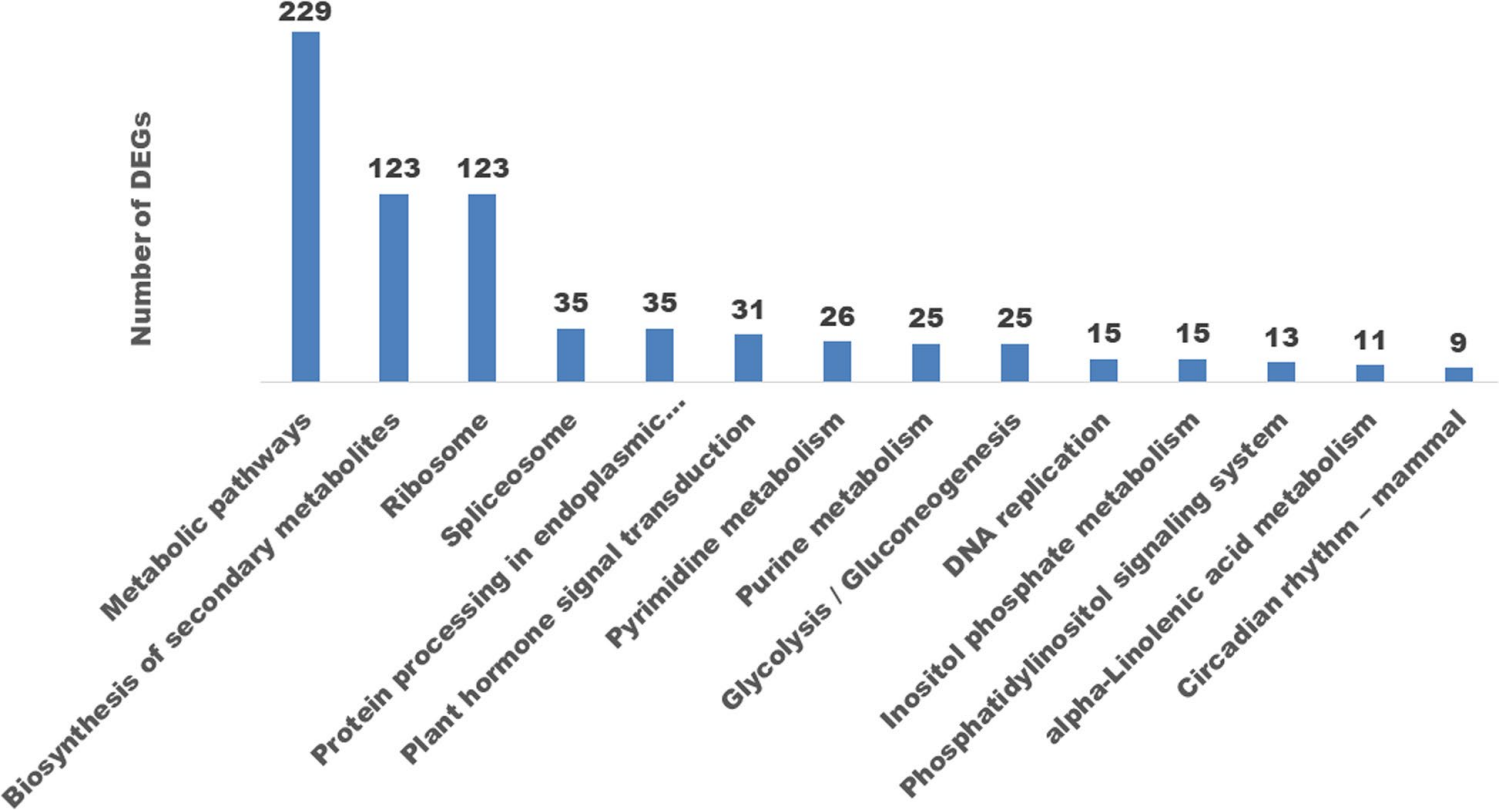
KEGG pathways enriched between *ny1* and H1 (WT) during meiosis

A KEGG pathway enrichment analysis of DEGs was performed using the KEGG pathway database to identify the biological pathways between *ny1* and H1. A total 715 of 5606 DEGs between *ny1* and H1 were classified into 14 functional categories (https://www.kegg.jp/). As shown in Fig. 5, these transcripts were mainly involved in three KEGG pathways, including metabolic pathways, biosynthesis of secondary metabolites and ribosome.

KEGG pathway enrichment analysis of up- and down-regulated DEGs was also performed. A total of 305 and 371 up- and down-DEGs were found to have KEGG annotations, and 12 KEGG pathways were specially enriched in up-regulated, while 15 were enriched in the down-regulated DEGs, respectively (Figure S8a, b). Of these pathways, some important pathways related to fertility, including metabolic pathways, starch and sucrose metabolism, and peroxisome were detected in the down-regulated genes, while basal transcription factors was identified in the up- regulated DEGs.

Taking together, GO and KEGG analyses results showed that DEGs were involved in sucrose synthase, sucrose transporters, invertase, and hexokinase. Sucrose is decomposed into monosaccharides, which is transported into cells and used for starch biosynthesis [40, 41]. The sucrose synthase genes (*RSUS2* and *RSUS3*) and hexokinase genes (*OsHXK7 and OsHXK9*) were significantly down-regulated. Sucrose transporters (SUTs) are known to play critical roles in the sucrose uptake from the apoplast in various stages of sugar translocation [42, 43]. A sucrose transporter gene (*OsSUT1*) was found to be down-regulated between *ny1* and H1 (Table 4).

**Table 4:** Starch and sucrose metabolism related genes that displayed down regulation between *ny1* and H1

Name	ID	Description	*NY1*
*RSUS3*	*LOC_Os07g42490*	sucrose synthase gene	down
*RSUS2*	*LOC_Os06g09450*	sucrose synthase gene	down
*OsHXK7*	*LOC_Os05g09500*	hexokinase gene	down
*OsHXK9*	*LOC_Os01g52450*	hexokinase gene	down
*OsSUT1*	*LOC_Os03g07480*	sucrose transporter gene	down

Fatty alcohols and their derivatives are major components of the anther cuticle and pollen wall during pollen development in the flowering plants, which are rich in lipids [44–46]. The gene *DPW* (*LOC_Os03g07140*), associated with peroxisome pathway and a fatty alcohol synthesis gene for anther cuticle and pollen sporopollenin biosynthesis in rice, was found to be down-regulated between *ny1* and H1.

## *NY1* Defects alter expression of important genes associated with Tapetum and Meiosis in Neo-tetraploid Rice

**Fig. 6:**
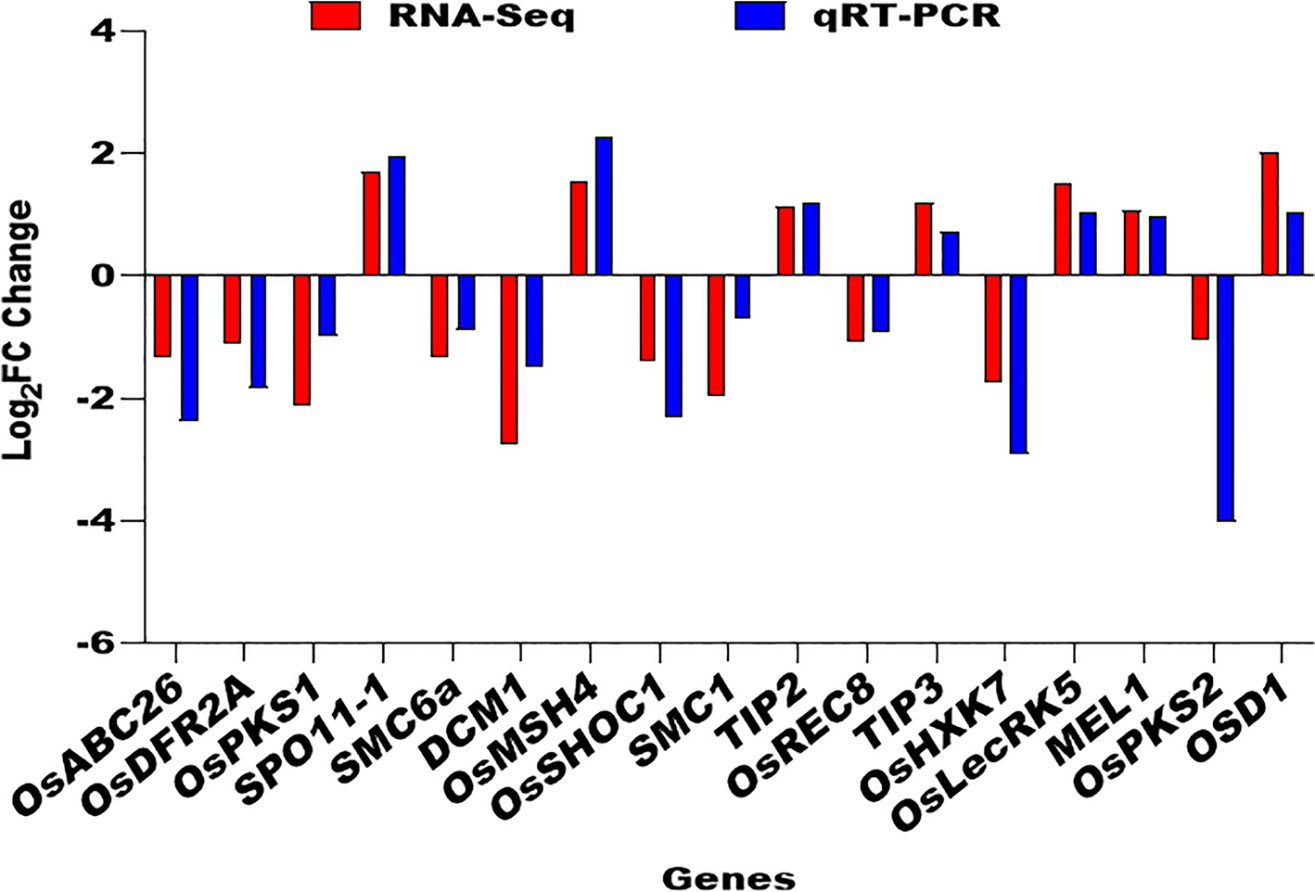
Comparison of the log_2_ (FC) of 17 selected genes using qRT-PCR analysis between *ny1* and H1

To further reveal the cause of low pollen fertility in *NY1*, we compared our differentially expressed genes (DEGs) between *ny1* and H1 with tapetum or pollen development genes reported in *Arabidopsis* and diploid rice [26, 27, 35]. We found that 31 genes were associated with tapetum or pollen development, including *EAT1, PTC1, CYP703A3, CYP704B2, DPW, OsABCG26, OsAGO2, SAW1, OsPKS1, OsPKS2,* and *OsTKPR1*. Among these genes, 15 were found to be up-regulated and 16 genes exhibited down-regulation between *ny1* and H1 (Table 5; Table S9). In order to verify the differential expression patterns of DEGs detected by RNA sequencing, a subset of 17 tapetum development and meiosis-related genes were selected for qRT-PCR validation during meiosis. Our results revealed that the expression trends of DEGs detected by qRT-PCR were consistent with the RNA-Seq analysis, with a correlation coefficient of R^2^ = 0.8096, thereby confirming the accuracy of the RNA sequencing results obtained in this study (Fig. 6; Figure S9).

**Table 5:** Downstream genes associated with tapetum development between *ny1* and H1

Gene name	RGAP ID	Protein feature	*P* value
*EAT1*	*Os04g0599300*	Basic helix-loop-helix transcription factor	6.53E-12
*OsTKPR1*	*Os09g0493500*	Tetraketide α-pyrone reductase	1.49E-44
*OsDFR2A*	*Os09g0493500*	NAD-dependent epimerase/dehydratase family protein	0.004661
*OsPKS2*	*Os07g0411300*	Plant-specific type III polyketide synthase	4.8E-88
*OsGL1-4*	*Os02g0621300*	Waxy synthetic gene	0.000
*CYP704B2*	*Os03g0168600*	CytochromeP450 protein	1.31E-24
*OsALDH2b1*	*Os06g0270900*	Aldehyde dehydrogenase	2.6E-219
*MADS3*	*Os01g0201700*	MADS-box gene	2.8E-187
*DLC1*	*Os02g0182100*	B-type response regulator gene	4E-102
*OsABCG26*	*Os10g0494300*	ATP Binding Cassette G26	8.4E-155
*SAW1*	*Os06g0638000*	CCCH-type zinc finger protein; defective callose in meiosis I	0.000
*DPW*	*Os03g0167600*	Fatty Acyl Carrier Protein Reductase	4.69E-10
*PTC1*	*Os09g0449000*	PERSISTENT TAPETAL CELL1	7.13E-30
*CYP703A3*	*Os08g0131100*	Cytochrome P450 hydroxylase	1.12E-44
*OsAGO2*	*Os04g0615700*	ARGONAUTE family protein	8.05E-50
*OsPKS1*	*Os10g0484800*	Polyketide synthase	3.03E-30

In addition, we further compared the 5606 differentially expressed genes (DEGs) between *ny1* and H1 (Table S4) with the meiosis-related genes reported in *Arabidopsis,* diploid and polyploid rice [10, 13, 26, 33, 35, 47]. A total of 1041 differentially expressed genes were meiosis-related genes, including 563 up- and 478 down-regulated genes (Table S10). Of these genes, a total of 29 important genes associated with meiosis were detected, including *OsREC8, OsRR24, OsSHOC1, DCM1, SMC1, SMC6a OsRAD17, OsRAD51A1, PAIR1, PAIR2* and *PSS1*. Among these genes, 20 were up- and 9 was down-regulated between *ny1* and H1 (Table S9).

### Predicted protein–protein interaction analysis of down-regulated DEGs associated with meiosis and tapetum

The predicted protein–protein interactions of 478 meiosis-stage-specific and meiosis-related genes were performed using STRING. We detected that the meiosis-specific gene, *OsPKS1* (*LOC_Os10g34360*), encodes a polyketide synthase, interacted with a meiosis-specific and a meiosis-related gene, including AMP-binding enzyme gene (*LOC_Os10g42800*) and a dihydroflavonol-4-reductase gene (*LOC_Os10g42620*). The meiosis-related gene *OsABCG26* (*LOC_Os10g35180*), is an ABC transporter, which interacted with AMP-binding enzyme genes (*LOC_Os11g35400*), universal stress protein domain containing protein (*LOC_Os10g30150*) and a drought tolerance gene (*LOC_Os11g10590*). Other important interactions were associated with eukaryotic translation initiation factor 4G, Acetyl-CoA carboxylase, and glycosyl hydrolase. All of these interactions suggest that the down-regulation of meiotic anther specific genes have a significant impact on the expression of important tapetum and meiosis-related or meiosis-specific genes (Figure S10).

### Gene knock-out of EAT1 (down-regulated) in neo-tetraploid rice

**Fig. 7:**
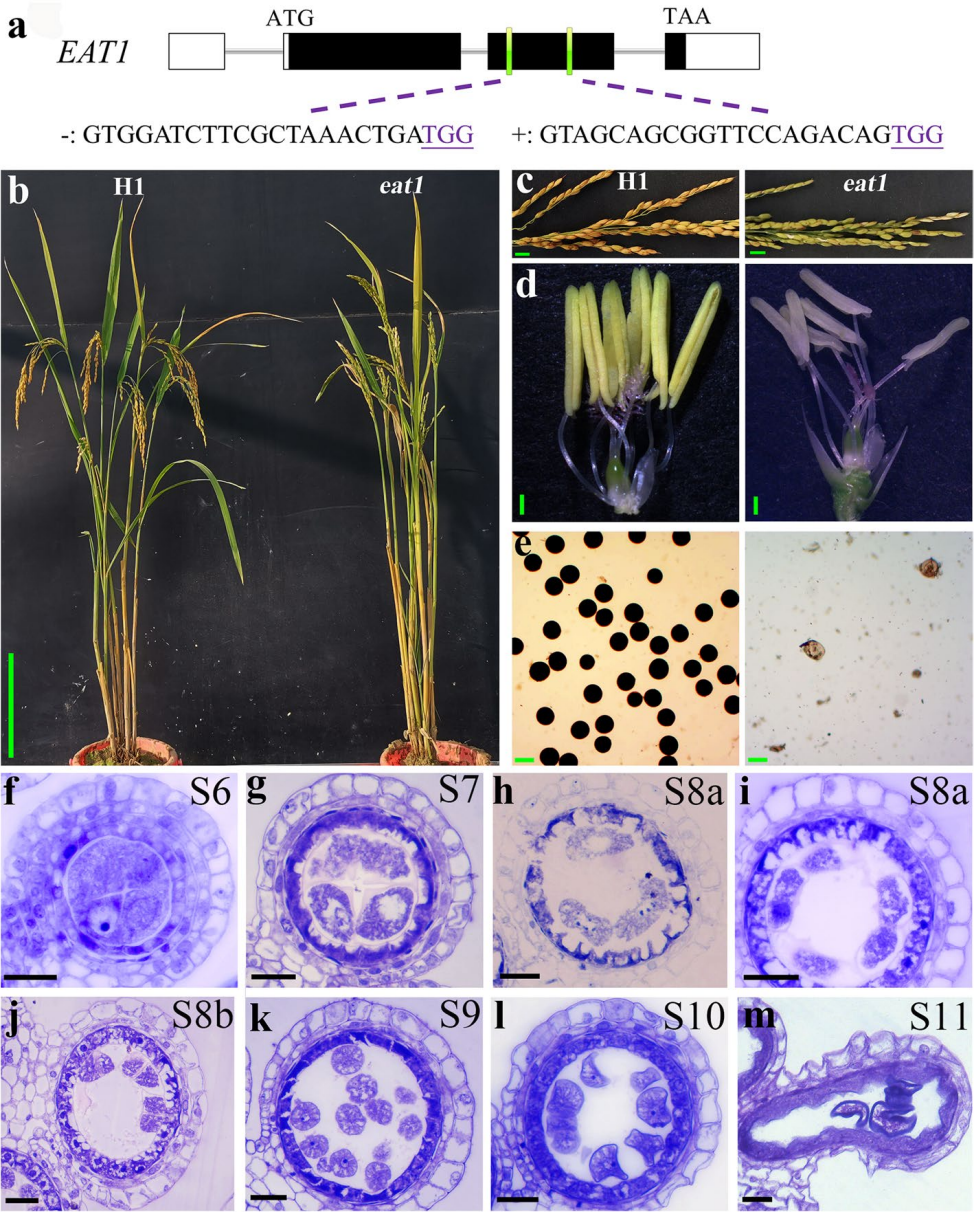
Phenotypic comparison and developing rice anther between H1 and *ny1*-related DEG mutant *eat1*. **a** Gene structure and CRISPR/Cas9 targets of *EAT1*. White areas, black areas, grey lines indicate untranslated regions (UTR), exons and introns, respectively. “ + ”, “-” indicated forward chain and reverse chain, respectively, where CRISPR/Cas9 target is located. The underline bases represent PAM (protospacer adjacent motif) for CRISPR/Cas9. **b** The plant type of H1 (WT) and *eat1*. Bars = 20 cm. **c** Mature panicles of H1 and *eat1*. Bars = 1 cm. **d** Floral organs of H1 and *eat1*. Bars = 0.5 mm. **e** I_2_-KI staining of H1 and *eat1* pollen grains at mature stage. Green bars = 50 μm. (f-m) Anther transverse section analysis of *eat1* in S6 **f**, S7 **g**, S8a **h**, **i**, S8b **j**, S9 **k**, S10 **l**, S11 **m**. Bars = 20 μm

To understand the reproductive relationship between *NY1* and its downstream gene *EAT1*, *EAT1* was edited by CRISPR/Cas9 system in neo-tetraploid line H1, since *EAT1* is involved in the pollen development of tetraploid rice (Fig. 7a). A total of 20 T_0_ positive transgenic plants were obtained, in which 80% of the T_0_ *eat1* plants showed complete sterility (0.00% seed setting, Fig. 7b-c and Table S11). The *eat1* had smaller and pale color anthers compared to H1 plants and produced non-viable pollens (Fig. 7d-e). In the anther transverse section analysis, the *eat1* anthers showed normal pollen mother cells (PMCs) and somatic layers (namely epidermis, endothecium, middle layer, and tapetum) at stage S6, just like the anthers of H1 (Fig. 7f). The tapetum cells showed delayed degeneration relative to H1, while the pollen mother cells could form tetrads during stage 8 (S8a and S8b) in *eat1* (Fig. 7g-j). Similar to *ny1*, the *eat1* tapetum cells were darkly stained and kept a very thick style, and the microspores were less stained and sporopollenin missing from stage 9 to stage 11, while the H1 tapetum cells would condense cytoplasm and became thinner (Fig. 7k-m). Therefore, these results together showed that the mutations of *NY1* and *EAT1* cause similar reproductive defects during tapetum degeneration.

## Discussion

### *NY1* may affect the gene expression network associated with tapetum development and meiosis in tetraploid rice

The plant pollen wall plays crucial roles in the development of pollen, and mature pollen is released from anther dehiscence. Dysfunction in the pollen cell wall could induce male sterility [48–52]. Abnormal development of anther cuticle cause defects in pollen development (48). Sporopollenin precursors produced in the tapetum can be transported onto the primexine for exine formation. Tapetum supply important materials for pollen wall formation, which ultimately effect pollen maturation and microspores. Defective pollen wall produces the majority of sterile pollens [49]. As a result, pollen growth is dependent on the synthesis and breakdown of these cell walls [50]. In this study, GO analysis revealed that biological processes related to cellular lipid catabolic process, cellular lipid metabolic process, cell death, and programmed cell death were significantly enriched in *ny1*. These results revealed that *ny1* is important for tapetum development. Sixteen down-regulated genes related to tapetum or pollen development were identified in *ny1*. In particular, these genes have previously been found to be down-regulated in tetraploid rice compared to diploid rice [10, 13, 16]. During rice anther development, the functions of these 16 genes have been extensively explored. For example, *PTC1* and *EAT1* contribute to tapetal cell death and pollen development [25, 53], while *CYP704B2* and *CYP703A3* regulate 7-hydroxylated lauric acid production and hydroxylation of fatty acids, both of them are essential for anther cutin biosynthesis and pollen exine creation in rice [54, 55]. *DPW* is a fatty alcohol synthesis gene that is involved in the formation of anther cuticle and pollen sporopollenin [56], while *OsABCG26* is an ABC transporter that is necessary for the production of pollen exine and anther cuticle, as well as pollen-pistil interactions [57]. *OsAGO2* promotes tapetal programmed cell death (PCD) and regulates ROS generation in rice [58]. *SAW1* is a new CCCH-type zinc finger protein that regulates gibberellin homeostasis and anther development in rice by activating *OsGA20ox3* [59], while *OsPKS1* is required for optimal pollen exine production [60]. *OsPKS2* participate in pollen wall production, which is necessary for rice male fertility [61], while *OsTKPR1* is involved in anther cuticle development and pollen wall creation in rice [62]. The loss of function of these genes promotes pollen sterility and an aberrant programmed cell death mechanism in tapetal cells in rice. Tapetal cell abnormalities were commonly detected in autotetraploid rice and may be the principal cause of reduced pollen fertility [13, 18]. In *ny1* mutant, a similar phenomenon of aberrant tapetal cells and defective pollen formation was also observed.

Meiosis plays important role in the rice pollen development [33]. More than 5000 meiosis-related genes have been identified in Arabidopsis, diploid and polyploid rice [9, 10, 13, 15, 26–35, 63]. The dysfunction of gene products associated with meiosis induced male sterility [64–66]. Many studies have shown that the down-regulation of meiosis-related and pollen fertility genes were the main reason for low fertility in autotetraploid rice [9, 12, 13, 29]. In the present study, we detected 478 meiosis-related genes that displayed significant down-regulation between *ny1* and H1. Among these genes, 9 important meiosis-related genes were identified, including *OsREC8* (*LOC_Os05g50410*) also known as *OsRad21-4*, is a chromosome structure maintenance protein, that is vital for chromatid cohesion and metaphase I monopolar orientation in rice meiosis [67]. *OsRR24* (*LOC_Os02g08500*) also known as *LEPTO1*, encodes a Type B response regulator that is essential for the organization of leptotene chromosomes in rice meiosis [68]. *OsSHOC1* (*LOC_Os02g42910*) encodes expressed protein that is essential for crossover formation during rice meiosis [69]. *MRE11* (*LOC_Os08g08030*) is required for homologous synapsis and double-strand break (DSB) processing in rice meiosis [70]. *DCM1* (*LOC_Os06g43120*) also known as *SAW1*, which encodes zinc finger protein and required for male meiotic cytokinesis by preserving callose in rice [71]. Knock-out mutant of these genes resulted in abnormal meiosis process and pollen sterility. Similar trend of abnormal meiosis process and pollen sterility was also observed in *ny1* mutant [23]. Taken together, these results suggest that the down-regulation of important tapetal development and meiosis-related genes play crucial role and might be a major reason for pollen sterility in *ny1* mutant.

### *NY1* and its downstream gene, EAT1, regulate tapetum development in tetraploid rice

Tapetal programmed cell death (PCD) is vital for pollen development, which has been controlled by a series of tapetal PCD development processes in rice [25, 51, 56]. After meiosis, Tapetum provide signaling molecules and nutrients to facilitate pollen development in rice, which undergoes PCD triggered cellular degradation [24, 72]. Tapetum development and later degeneration occur together with post meiotic events, and delayed or premature tapetal PCD often cause male sterility in plants [25, 50, 56, 73]. The male sterile mutants, *ptc1* (25) and *ptc2* (51) exhibited delayed tapetal PCD, which was revealed by ultra-thin sectioning in rice. In this study, we reported a novel rice gene, *NY1*, which is critical for pollen development. The *ny1* mutant showed delayed degradation of tapetal and middle wall cell layers, the microspores were less stained and lack of sporopollenin, and aborted mature pollens compared to its wild type (H1).

In *eat1* mutant, the tapetum cells showed more delayed degeneration relative to H1, while the pollen mother cells could form tetrads during stage 8 (S8a and S8b) in *eat1*. Similar to *ny1*, the *eat1* tapetum cells were darkly stained and kept a very thick style, and the microspores were less stained and lack of sporopollenin from stage 9 to stage 11, while the H1 tapetum cells would condense cytoplasm and became thinner. These results together showed that the mutations of *NY1* and *EAT1* cause similar reproductive defects during tapetum degeneration, which ultimately lead to partial pollen abortion in mutants.

### Changes in carbohydrate metabolism related genes expression may cause partial pollen sterility in *ny1* mutant

Carbohydrates play a vital role in the development of pollen and anthers [74], acting as an energy source for developing anthers and pollen [75]. Pollen sterility can be caused by changes in carbohydrate metabolism or the supply of assimilate [76]. The combined regulatory process involves a large number of related genes or proteins. Previous research has found that aberrant gene expression in anthers disrupts pollen formation and decrease pollen fertility. Changes in enzyme and carbohydrate activity expression could also reduce sugar and starch accumulation in the anthers [77]. Here, large numbers of DEGs in the KEGG pathways were enriched in metabolic pathways and starch and sucrose metabolism. These two pathways have been reported to play important roles in anther and pollen development in rice [12, 16].

The supply of carbohydrates from the leaves to pollen grain involves sucrose transport and degradation, monosaccharides formation and transport, and starch generation [41, 43]. There are two types of enzymes that catalyze the sucrose degradation in plants, one is invertase and the other is sucrose synthase (SUS) [40]. In our study, the sucrose synthase genes (*RSUS2* and *RSUS3)* were down regulated and the invertase (*OsINV4*) was found to be up-regulated between *ny1* and H1. After sucrose degradation, the resulting hexoses undergo phosphorylation by hexokinase for starch synthesis. Hexose serves as an energy source, a compatible solute for pollen formation, and a substance for cell wall synthesis. In rice, deficiency of hexokinase *HXK5* impairs synthesis and utilization of starch in pollen grains and causes male sterility [78]. The hexokinase 10 (*OsHXK10*) RNAi lines are male-sterile, probably due to a defect in anther dehiscence in rice [79]. Here, hexokinase genes, *OsHXK7* and *OsHXK9*, were found to be down-regulated, while *OsHXK1* and *OsHXK10* displayed up-regulation between *ny1* and H1. Heterotrophic cells, such as roots and seeds, are sink organs and rely on the supply of sugars for their nutrition. Thus, the adequate production, storage and transport of sugars are essential to sustain plants growth and development. Lemoine et al. [80] identified a pollen-specific sucrose transporter (*NtSUT3*) in tobacco that regulates pollen development and supplies nutrition to pollen tubes. The deletions of *OsSUT1* gene disrupt pollen function in rice [42]. Here, the same sucrose transporter gene (*OsSUT1*) was found to be down-regulated between *ny1* and H1. The loss of function of these genes resulted in male sterility of their mutant plants. A similar phenomenon of male sterility was also observed in *ny1* mutant.

## Conclusions

In this study, the cytological observation of the *ny1* and its downstream gene *eat1*, exhibited abnormal tapetum and pollen wall formation. Furthermore, the abrupt changes in expression of tapetal, meiosis and carbohydrate metabolisms related genes might responsible for the partial pollen sterility in *ny1*. Given the importance of rice as a major crop, this finding may provide molecular basis for rational manipulation of the 478 down-regulated meiosis-related candidate transcripts to improve rice yield, especially seed setting of polyploid rice.

## Methods

### Plant materials and growing conditions

Three types of materials were used in this study, which included wild type Huaduo 1 (H1), and *ny1* and *eat1* mutants. H1 is a neo-tetraploid rice variety with high fertility, which developed by our research team and was used as a control (WT). The *ny1* was created using CRISPR/Cas9 system by knock-out *NY1*, and *eat1* by knock-out of *EAT1* (For CRISPR/Cas9 method, please refer to the section “development and identification of eat1 mutant plants in huaduo1”). All materials were grown at the farm of South China Agricultural University (Guangzhou: 23_N, 113_E, Guangdong) under natural conditions and managed according to the recommended protocol for the area.

### Pollen fertility and semi-thin section analysis

The pollen fertility was observed according to our previous study with minor modifications [21]. The mature pollen grains were observed by staining with 1% I_2_-KI under a microscope (Motic BA200, China). Ten plants were randomly selected to observe the pollen fertility of wild type, mutant (*ny1*) and their F_1_ hybrids.

Semi-thin section analysis was performed according to Li et al. [13]. The anthers at different pollen development stages were collected and fixed in formalin-acetic acid-alcohol (FAA) solution for 48 h at room temperature. After dehydration through an ethanol series, tissues were embedded in Technovit 7100 histologische untersuchungen (Mikrotomschnitte Weichgewebe) according to the manufacturers’ protocol (Heraeus Kulzer). The embedded samples were further sectioned using a Leica RM2235 manual rotary microtome, stained with 1% toluidine blue O, and sealed with neutral balsam. The detailed procedures were described previously [13].

### Evaluation of seed setting rate

Seed setting rate of the F_1_ hybrids, F_2_ and their parents were investigated at maturity. The standard for investigating these traits was according to the protocols of People’s Republic of China for the registration of a new plant variety Distinctness, Uniformity and Stability (DUS) test guidelines of rice (Guidelines for the DUS test in China, 2012) [15]. We preformed one-way analysis of variance (ANOVA) and Duncan’s multiple range test (DMRT) to identify significant (*p* < 0.05) differences between group averages, using the SPSS 19.0 statistical software.

### RNA-seq experiments and data analysis

The anthers of T_3_ transgenic lines of *ny1* (homozygous mutants) and H1 (control) at the meiotic stage (Table S14) were collected in three biological replicates and stored at − 80 °C for RNA isolation. Total RNA was taken according to the manual instructions of the TRIzol Reagent (Life technologies, California, USA). The RNA-seq process was performed according to a previously described approach [12]. The gene expression differences between samples were detected using the DESeq package. The DEGs were identified with FDR (false discovery rate) ≤ 0.05 and the absolute value of log2 (Fold change) ≥ 1, and then DEGs were used for subsequent analysis. GO enrichment analysis was conducted using agriGO 2.0 (Beijing, China). The KEGG database was used to determine the metabolic pathways associated with differentially expressed genes [81]. Predicted protein–protein interactions were analyzed using STRING website (http://www.string-db.org/).

### Real-time quantitative polymerase chain reaction (qRT-PCR) assay

The important differentially expressed genes (DEGs) were validated by qRT-PCR**.** The rice ubiquitin gene (*LOC_Os03g13170*) was selected and used as an internal control to normalize the expression levels and all primers for qRT-PCR were designed by Primer Premier 5.0 and Primer-Blast software in NCBI (Table S13). All qRT-PCR reactions were performed in three biological replicates, and the results were calculated using the 2^−ΔΔCt^ method [82].

### Development and identification of eat1 mutant plants in Huaduo1

CRISPR/Cas9 system was used to generate mutation of candidate gene as previously reported [83]. The two targets were designed for the candidate gene to obtain single guide RNA (sgRNA) expression cassettes (U6a and U6b promoters), which were incorporated into the CRISPR/Cas9 vector pLYCRISPR/Cas9Pubi-H (Table S12). Then, the vectors were transferred into Huaduo1 (H1). The target region for the mutant was amplified by PCR, and the segment was subjected to Sanger sequencing. Transgenic seedlings were examined under natural field condition at the experimental farm of South China Agriculture University, Guangzhou, China. The T_2_ plants of homozygous mutants were used for phenotypic and genotypic analyses (Table S11).

## Supplementary Information

**Additional file 1:** **Figure S1**. Analysis of variations in *NY1* sequence of 121 rice materials. **Figure S2**. Prediction of tertiary structure of mutant proteins. **Figure S3**. Cluster analysis of *NY1* in 121 rice materials. **Figure S4**. Significant GO terms of up-regulated differentially expressed genes in molecular function category between *ny1* and H1 (WT) during meiosis. **Figure S5**. Significant GO terms of down-regulated differentially expressed genes in molecular function category between *ny1* and H1 (WT) during meiosis. **Figure S6**. Significant GO terms of up-regulated differentially expressed genes in cellular component category between *ny1*and H1 (WT) during meiosis. **Figure S7**. Significant GO terms of down-regulated differentially expressed genes in cellular component category between *ny1 *and H1 (WT) during meiosis. **Figure S8**. KEGG pathways enriched between *ny1 *and H1 (WT) during meiosis. (a) Down regulated pathways DEGs. (b) Up regulated pathways DEGs. **Figure S9**. Comparison of the log2 (FC) of 17 selected genes using qRT-PCR analysis between *ny1* and H1. **Figure S10**. Predicted protein-protein interaction network of meiosis and tapetum and meiosis-related or meiosis-specific genes associated with down-regulated DEGs.

**Additional file 2:** **Table S1**. Mutations detected in the *NY1* sequence of 121 rice materials. **Table S2. **Analysis of *NY1* DNA sequence variations in neo-tetraploid (H3) compared to autotetraploid (T452) rice. **Table S3-1 to S3-24.** Mutation site of *ny1 *allele, reference allele and the alternative allele type1 to 22.

**Additional file 3:** **Table S4.** Pollen fertility and seed setting of F_2_ populations, WT (H1) and *ny1* mutant**. Table S5**. Overview of quality reads between *ny1* and H1 (WT) during meiosis. **Table S6**. Pearson correlation analysis of H1 (WT) compared with *ny1*. **Table S7.** Differentially expressed genes between *ny1* and H1 (WT) during meiosis. **Table S8a**. Significant up-regulated GO terms of differentially expressed genes between *ny1* and H1 during meiosis. **Table S8b**. Significant down-regulated GO terms of differentially expressed genes between *ny1* and H1 during meiosis.** Table S9**. Known tapetum and meiosis-related genes detected during meiosis between *ny1* and H1 (WT).** Table S10. **Meiosis-related and stage-specific genes detected during meiosis between *ny1* and H1.** Table S11**. The seed setting and mutant information of H1 (WT) and *eat1.* **Table S12**. guide RNA information of *eat1.* **Table S13**. List of primers used for qRT-PCR analysis. **TableS14**. Floret length during meiosis in H1 (WT) and *ny1* (mutant) rice.

## Data Availability

All data supporting the conclusions described here are provided in tables, figures and additional files.

## Abbreviations

BSA: Bulked segregant analysis
DEGs: Differentially expressed genes
qRT-PCR: Quantitative real-time polymerase chain reaction
PCD: Programmed cell death
PMCs: Pollen mother cells
Leica: SPE Laser scanning confocal microscope
WE-CLSM: Whole-mount eosin B-staining confocal laser scanning microscopy
WT: Wild-type
