# Supplementary material for: An uncharacterized protein NY1 targets EAT1 to regulate anther tapetum development in polyploid rice
Source: BMC Plant Biol. 2022 Dec 13;22:582. doi: 10.1186/s12870-022-03976-0 (PMC9746164; doi:10.1186/s12870-022-03976-0)
Supplement: Supplementary file 1 — Additional file 1: Figure S1. Analysis of variations in NY1 sequence of 121 rice materials. Figure S2. Prediction of tertiary structure of mutant proteins. Figure S3. Cluster analysis of NY1 in 121 rice materials. Figure S4. Significant GO terms of up-regulated differentially expressed genes in molecular function category between ny1 and H1 (WT) during meiosis. Figure S5. Significant GO terms of down-regulated differentially expressed genes in molecular function category between ny1 and H1 (WT) during meiosis. Figure S6. Significant GO terms of up-regulated differentially expressed genes in cellular component category between ny1and H1 (WT) during meiosis. Figure S7. Significant GO terms of down-regulated differentially expressed genes in cellular component category between ny1 and H1 (WT) during meiosis. Figure S8. KEGG pathways enriched between ny1 and H1 (WT) during meiosis. (a) Down regulated pathways DEGs. (b) Up regulated pathways DEGs. Figure S9. Comparison of the log2 (FC) of 17 selected genes using qRT-PCR analysis between ny1 and H1. Figure S10. Predicted protein-protein interaction network of meiosis and tapetum and meiosis-related or meiosis-specific genes associated with down-regulated DEGs. [file 12870_2022_3976_MOESM1_ESM.docx]

**Additional files:**

**Additional file 1: Figure S1**. Analysis of variations in *NY1* sequence of 121 rice materials. **Figure S2**. Prediction of tertiary structure of mutant proteins. **Figure S3**. Cluster analysis of *NY1* in 121 rice materials. **Figure S4**. Significantly GO terms of up-regulated differentially expressed genes in molecular function category between *ny1* and H1 (WT) during meiosis. **Figure S5**. Significantly GO terms of down-regulated differentially expressed genes in molecular function category between *ny1* and H1 (WT) during meiosis. **Figure S6**. Significantly GO terms of up-regulated differentially expressed genes in cellular component category between *ny1* and H1 (WT) during meiosis. **Figure S7**. Significantly GO terms of down-regulated differentially expressed genes in cellular component category between *ny1* and H1 (WT) during meiosis. **Figure S8**. KEGG pathways enriched between *ny1* and H1 (WT) during meiosis. (a) Down regulated pathways DEGs. (b) Up regulated pathways DEGs. **Figure S9**. Comparison of the log2 (FC) of 17 selected genes using qRT-PCR analysis between *ny1* and H1. **Figure S10**. Predicted protein-protein interaction network of meiosis and tapetum and meiosis-related or meiosis-specific genes associated with down-regulated DEGs. **Additional file 2: Table S1**. Mutations detected in the *NY1* sequence of 121 rice materials. **Table S2.** Analysis of NY1 DNA sequence variations in neo-tetraploid (H3) compared to autotetraploid (T452) rice. **Table S3-1 to S3-24.** Mutation site of *ny1* allele, reference allele and the alternative allele type1 to 22. **Additional file 3: Table S4.** Pollen fertility and seed setting of F_2_ populations, WT (H1) and *ny1* mutant**. Table S5**. Overview of quality reads between *ny1* and H1 (WT) during meiosis. **Table S6**. Pearson correlation analysis of H1 (WT) compared with *ny1*. **Table S7.** Differentially expressed genes between *ny1* and H1 (WT) during meiosis. **Table S8a**. Significant up-regulated GO term of differentially expressed genes between *ny1* and H1 during meiosis. **Table S8b**. Significant down-regulated GO term of differentially expressed genes between *ny1* and H1 during meiosis. **Table S9**. Known tapetum and meiosis-related genes detected during meiosis between *ny1* and H1 (WT). **Table S10.** Meiosis-related and stage-specific genes detected during meiosis between *ny1* and H1. **Table S11**. The seed setting and mutant information of H1 (WT) and *eat1.* **Table S12**. guide RNA information of *eat1.* **Table S13**. List of primers used for qRT-PCR analysis. **Table S14**. Floret length during meiosis in H1 (WT) and *ny1* (mutant) rice.


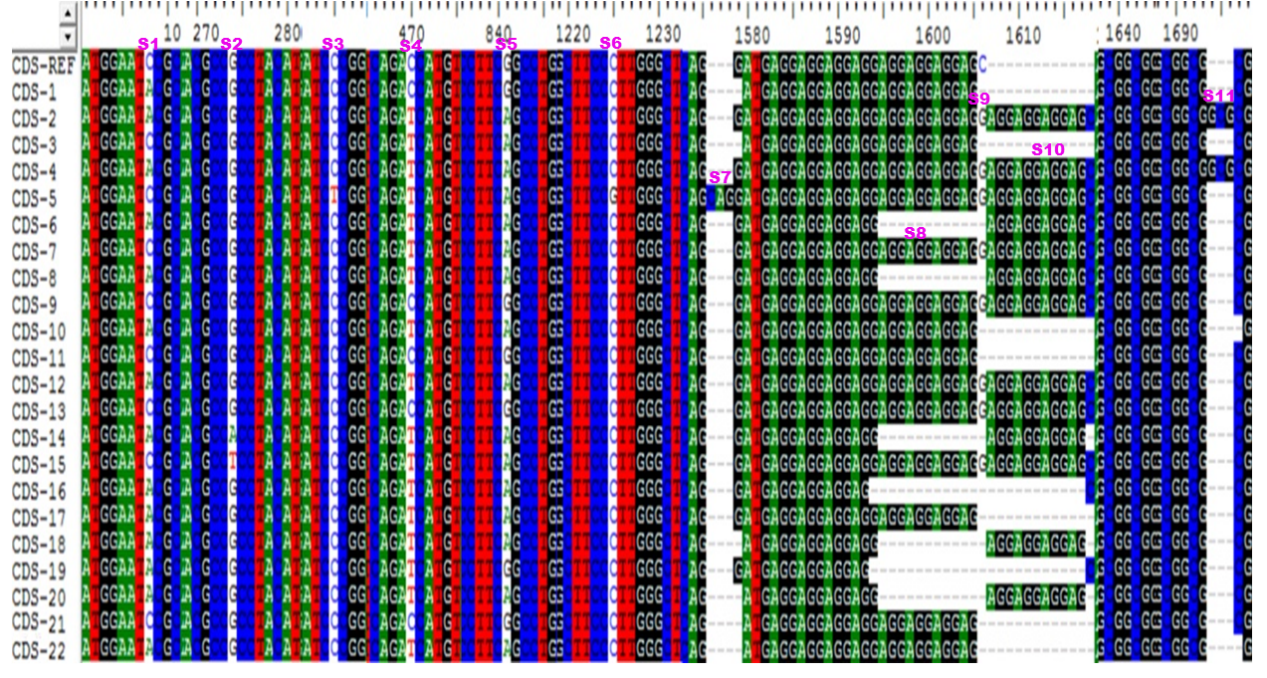
Additional file 1: Figure S1. Analysis of variations in *NY1* sequence of 121 rice materials

Note: S1-S11 (pink font) indicate 11 mutation sites, while CDS-1-22 represent mutation types compared to Nipponbare reference genome in different materials. Please see Table S1 for mutation types.


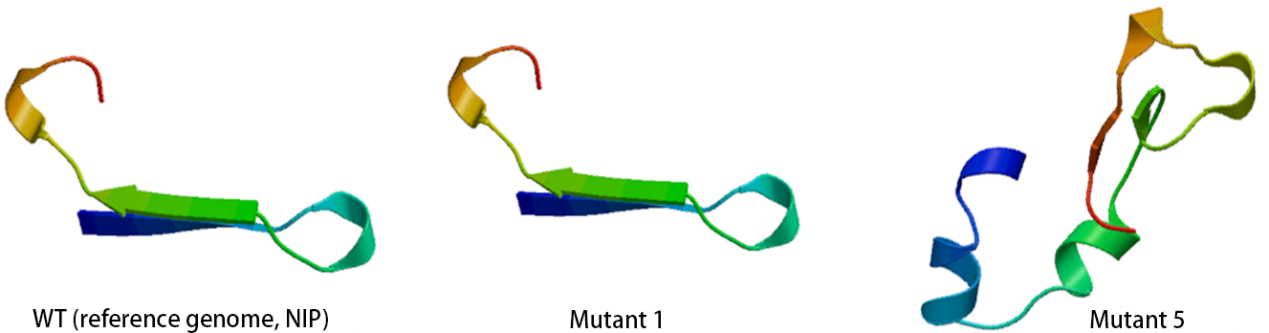


Additional file 1: Figure S2. Prediction of tertiary structure of mutant proteins


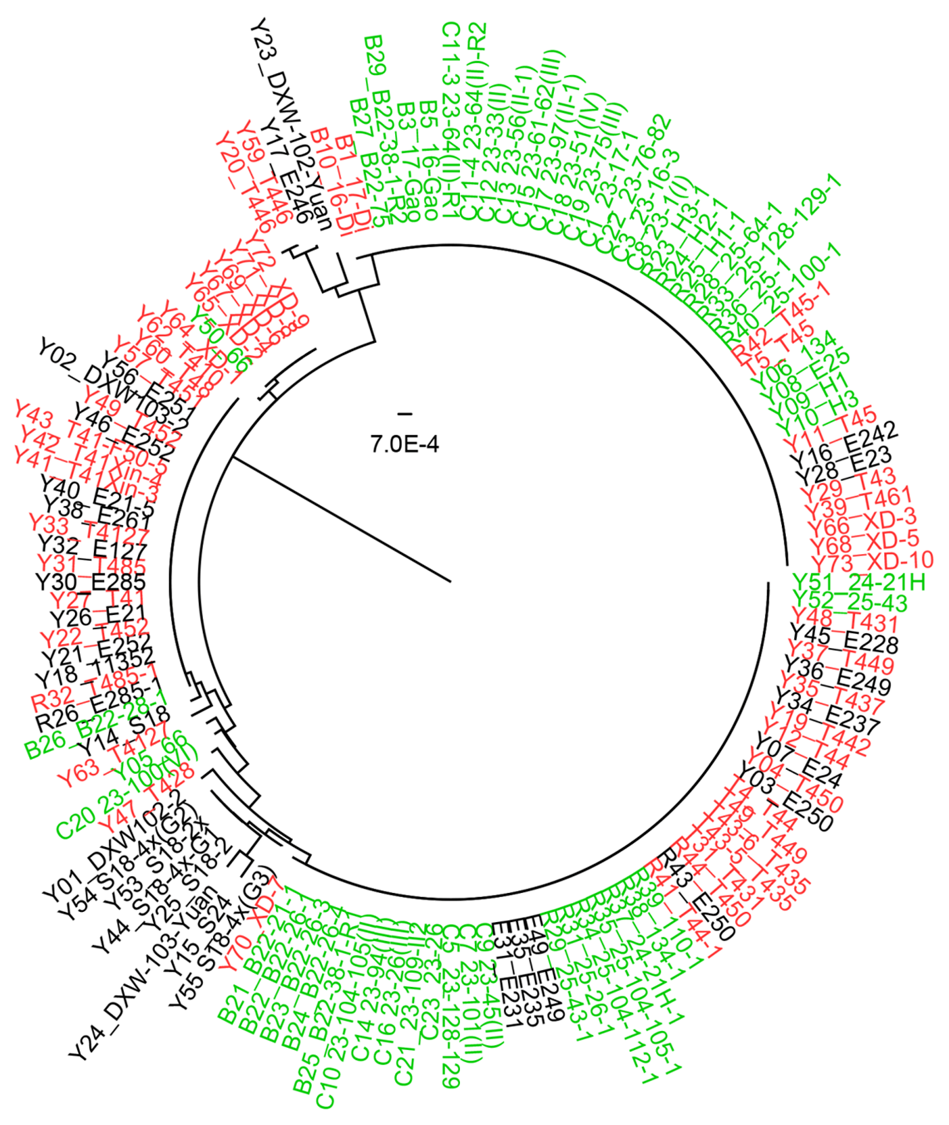


Additional file 1: Figure S3. Cluster analysis of *NY1* in 121 rice materials

The rice lines in green are neo- tetraploid lines with high fertility; the rice lines in pink are autotetraploid lines with low fertility; and the rice lines in black are diploid lines with high fertility.

Genome classification, population structure and types of some lines are mentioned in Yu et al. 2021 [21].


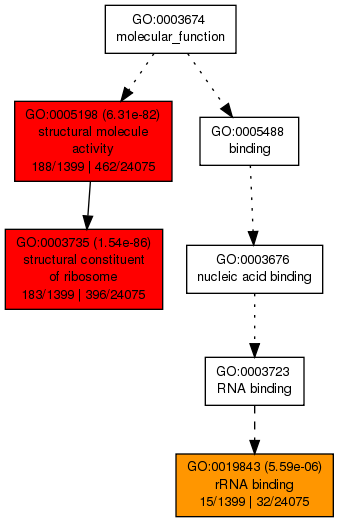


Additional file 1: Figure S4. Significantly up-regulated GO terms of differentially expressed genes in molecular function category between *ny1* and H1 (WT) during meiosis


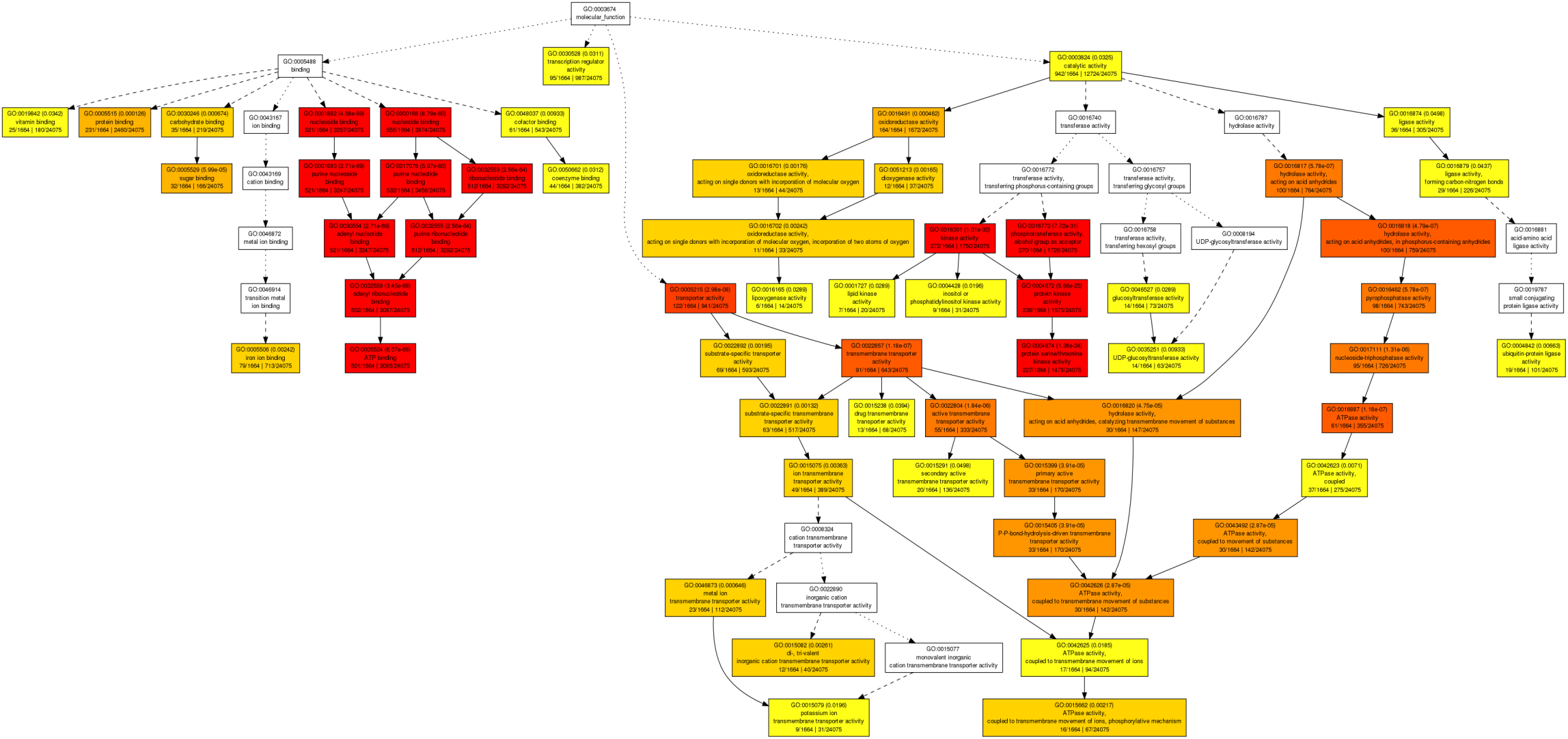


Additional file 1: Figure S5. Significantly down-regulated GO terms of differentially expressed genes in molecular function category between *ny1* and H1 (WT) during meiosis


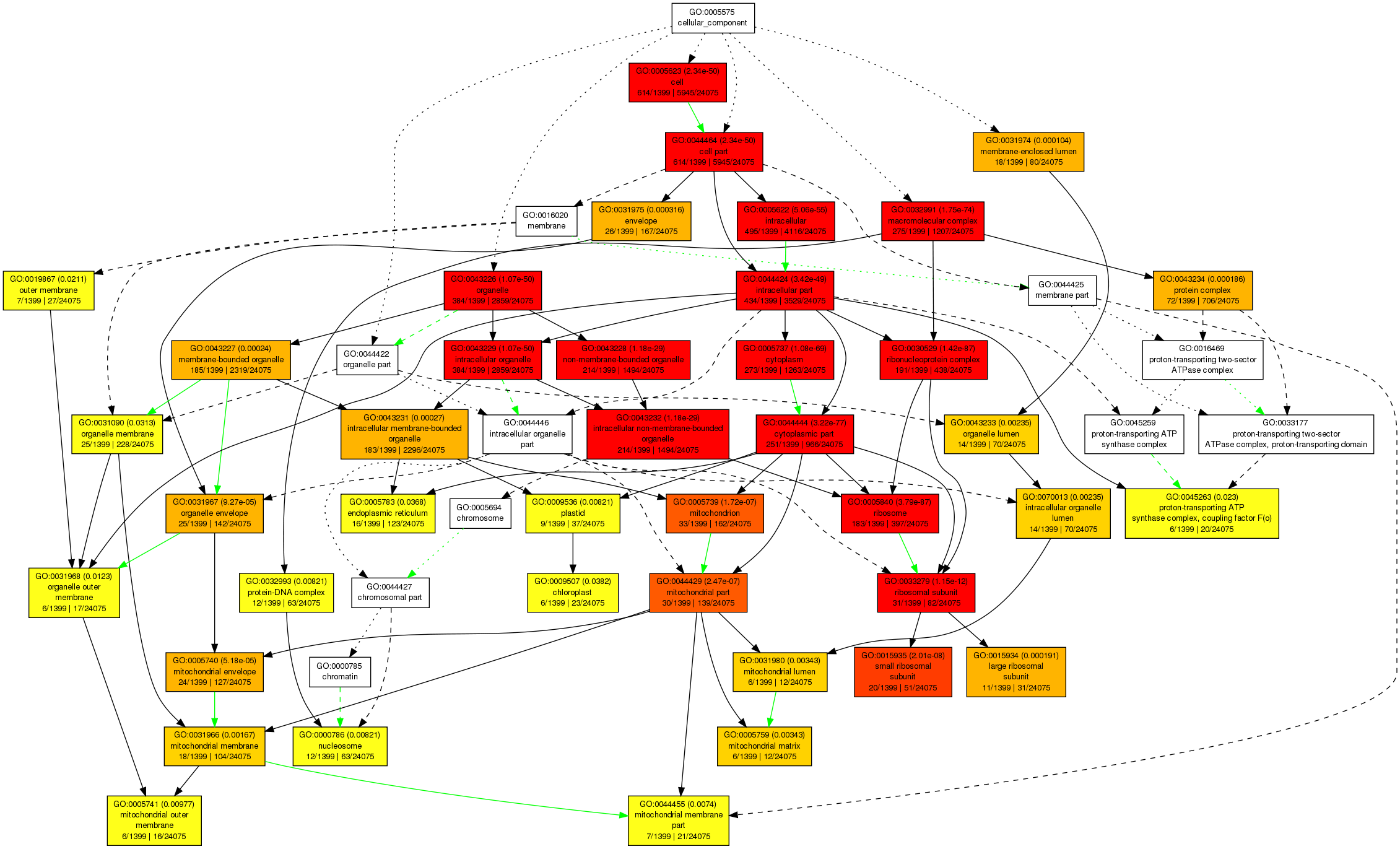


Additional file 1: Figure S6. Significantly up-regulated GO terms of differentially expressed genes in cellular component category between *ny1* and H1 (WT) during meiosis


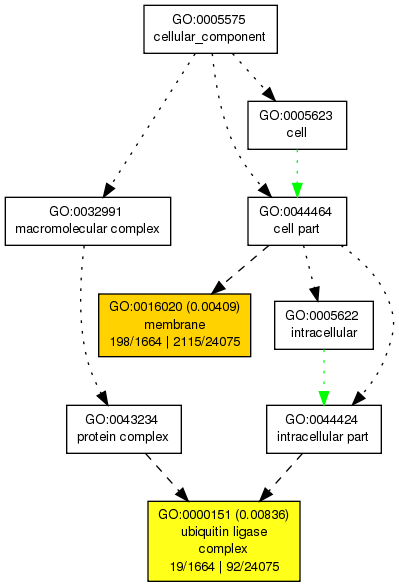


Additional file 1: Figure S7. Significantly down-regulated GO terms of differentially expressed genes in cellular component category between *ny1* and H1 (WT) during meiosis


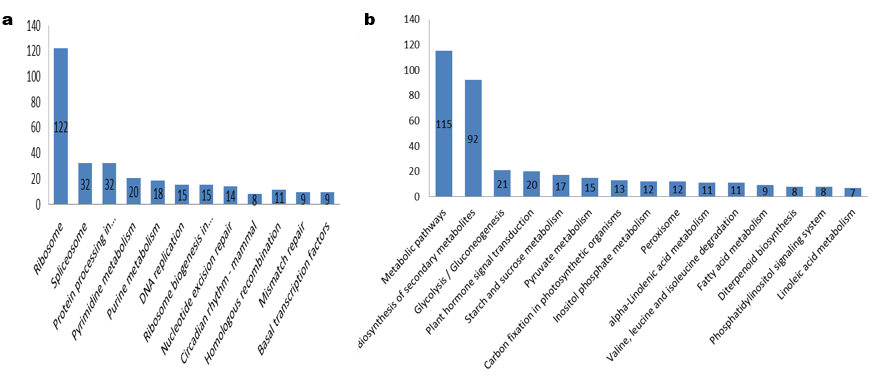
Additional file 1: Figure S8. KEGG pathways enriched between *ny1* and H1 (WT) during meiosis.

(a) Up-regulated pathways DEGs. (b) Down-regulated pathways DEGs


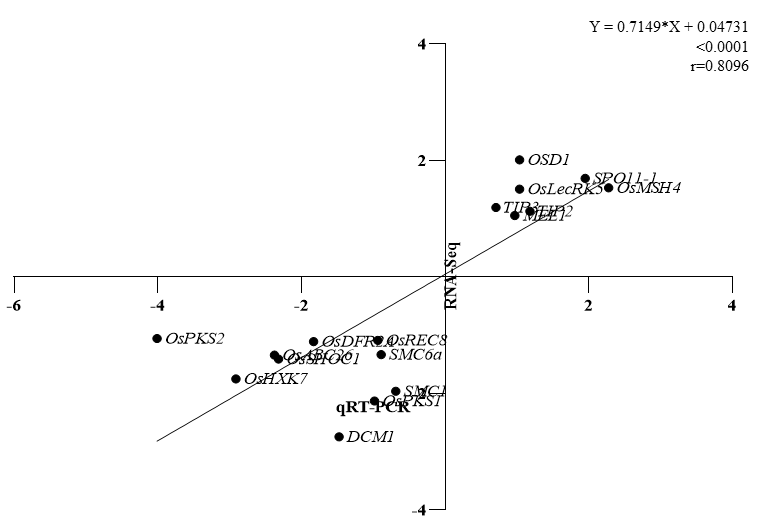


Additional file 1: Figure S9. Comparison of the log2 (FC) of 17 selected genes using qRT-PCR analysis between *ny1* and WT.

Note: Both the log2 transformed values of relative expression of qRT-PCR and fold changes of RNA seq data were used for linear regression. The Pearson’s correlation coefficient was used to measure the linear correlation between qRT-PCR and RNA seq analysis


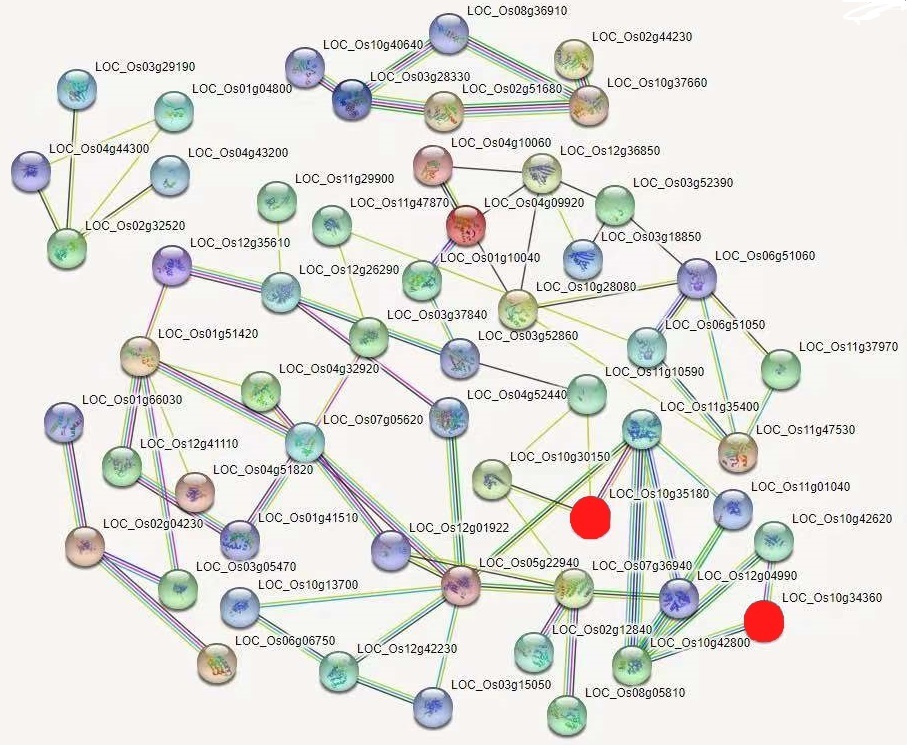


Additional file 1: Figure S10. Predicted protein-protein interaction network of meiosis and tapetum genes associated with down-regulated DEGs.

The protein-protein interaction sub-network was constructed using three important meiosis related and meiosis specific genes (red) and other genes that interact with these genes.
